# Absence of *Elovl6* attenuates steatohepatitis but promotes gallstone formation in a lithogenic diet-fed *Ldlr*^−/−^ mouse model

**DOI:** 10.1038/srep17604

**Published:** 2015-12-01

**Authors:** Motoko Kuba, Takashi Matsuzaka, Rie Matsumori, Ryo Saito, Naoko Kaga, Hikari Taka, Kei Ikehata, Naduki Okada, Takuya Kikuchi, Hiroshi Ohno, Song-iee Han, Yoshinori Takeuchi, Kazuto Kobayashi, Hitoshi Iwasaki, Shigeru Yatoh, Hiroaki Suzuki, Hirohito Sone, Naoya Yahagi, Yoji Arakawa, Tsutomu Fujimura, Yoshimi Nakagawa, Nobuhiro Yamada, Hitoshi Shimano

**Affiliations:** 1 Department of Internal Medicine (Endocrinology and Metabolism), Faculty of Medicine, University of Tsukuba, 1-1-1 Tennodai, Tsukuba, Ibaraki 305-8575, Japan; 2 International Institute for Integrative Sleep Medicine (WPI-IIIS), University of Tsukuba, Tsukuba, Ibaraki, Japan; 3 Laboratory of Proteomics and Biomolecular Science, Biomedical Research Center, Juntendo University Graduate School of Medicine, Tokyo 113-8421, Japan; 4 Faculty of Life and Environmental Sciences, University of Tsukuba, 1-1-1 Tennodai, Tsukuba, Ibaraki 305-8572, Japan; 5 Department of Internal Medicine, Faculty of Medicine, Niigata University, 1-754 Asahimachi, Niigata 951-8510, Japan

## Abstract

Nonalcoholic steatohepatitis (NASH) is a progressive form of nonalcoholic fatty liver disease (NAFLD) that can develop into liver cirrhosis and cancer. Elongation of very long chain fatty acids (ELOVL) family member 6 (Elovl6) is a microsomal enzyme that regulates the elongation of C12–16 saturated and monounsaturated fatty acids (FAs). We have previously shown that Elovl6 plays an important role in the development of hepatic insulin resistance and NASH by modifying FA composition. Recent studies have linked altered hepatic cholesterol homeostasis and cholesterol accumulation to the pathogenesis of NASH. In the present study, we further investigated the role of Elovl6 in the progression of lithogenic diet (LD)-induced steatohepatitis. We showed that the absence of Elovl6 suppresses hepatic lipid accumulation, plasma total cholesterol and total bile acid (BA) levels in LDL receptor-deficient (*Ldlr*^−/−^) mice challenged with a LD. The absence of Elovl6 also decreases hepatic inflammation, oxidative stress and liver injury, but increases the formation of cholesterol crystals in the less dilated gallbladder. These findings suggest that Elovl6-mediated changes in hepatic FA composition, especially oleic acid (C18:1n-9), control handling of hepatic cholesterol and BA, which protects against hepatotoxicity and steatohepatitis, but promotes gallstone formation in LD-fed *Ldlr*^−/−^ mice.

Currently, nonalcoholic fatty liver disease (NAFLD) is the most common form of chronic liver disease worldwide^1^,^2^,^3^,^4^. NAFLD encompasses a wide spectrum of conditions associated with the over-accumulation of lipids in the liver, ranging from steatosis to nonalcoholic steatohepatitis (NASH), which includes hepatitis and fibrosis. The mechanisms of disease progression in NASH are not yet completely understood.

It has been hypothesized that the development of NASH requires ‘two hits’^5^. The first hit is the development of hepatic steatosis and the second hit includes cellular stresses such as oxidative stress and elevated levels of proinflammatory cytokines. Recently, growing evidence suggests that simple steatosis and NASH may be two separate diseases. In this ‘multiple parallel hit’ hypothesis, the accumulated lipotoxic/proinflammatory lipid species interact with proinflammatory factors to cause progression to NASH; whereas, in other cases the liver develops steatosis and remains free of inflammation^6^,^7^. Therefore, it is important to identify toxic lipids. Although large epidemiological studies have suggested that triglyceride (TG)-mediated pathways may negatively affect NAFLD^8^, recent evidence indicates that TGs may, in fact, have a protective function^9^,^10^. Several studies have shown that the dysregulation of cholesterol metabolism is involved in the pathogenesis of NAFLD. Epidemiological data indicate a relationship between increased cholesterol intake and the risk and severity of NAFLD^11^,^12^. In addition, experimental models have demonstrated that free cholesterol loading precipitates steatohepatitis^13^.

The fatty acid (FA) composition of lipid species could be another determinant of the development of liver injury. Elongation of very long fatty acids (ELOVL) family member 6 (Elovl6) is a microsomal enzyme that regulates the elongation of C12–16 saturated and monounsaturated FAs^14^,^15^. The absence of Elovl6 function reduces stearate (C18:0) and oleate (C18:1n-9) levels and increases palmitate (C16:0) and palmitoleate (C16:1n-7) levels^16^. In our previous study, we reported that mice with a targeted disruption of Elovl6 (*Elovl6*^−/−^) were protected against the development of hepatic insulin resistance^16^, the deterioration of the insulin secretory function of pancreatic β-cells^17^ and macrophage foam cell formation^18^. Our findings suggest that the alteration of FA composition by Elovl6 deficiency not only affects lipid accumulation but also plays a vital role in a wide range of cellular functions and in disease progression.

Low density lipoprotein (LDL) receptor (LDLR)-deficient (*Ldlr*^−/−^) mice have been used in numerous studies as a model for atherosclerosis. It has been reported that *Ldlr*^−/−^ mice fed a high-cholesterol diet have increased sensitivity to inflammation, apoptosis and fibrosis of the liver^19^,^20^,^21^. In addition, *Ldlr*^−/−^ mice have a human-like lipoprotein profile^22^. Therefore, *Ldlr*^−/−^ mice can be used to study the early progression of NASH. We recently reported that atherogenic high-fat (AHF) diet-induced hepatic inflammation, oxidative damage and fibrosis in the liver were attenuated in *Elovl6*^−/−^ mice, despite comparable hepatosteatosis in *Elovl6*^−/−^ mice and wild-type mice^23^. To advance our understanding of the progression of NASH and to investigate the impact of Elovl6 deficiency on the development and progression of NASH, in the present study we used a lithogenic diet (LD) to induce NASH in *Ldlr*^−/−^ mice.

## Results

### Effects of Elovl6 deficiency on body and tissue weight alterations in LD-fed *Ldlr* ^**−**/**−**^ mice

**Figure 1:**
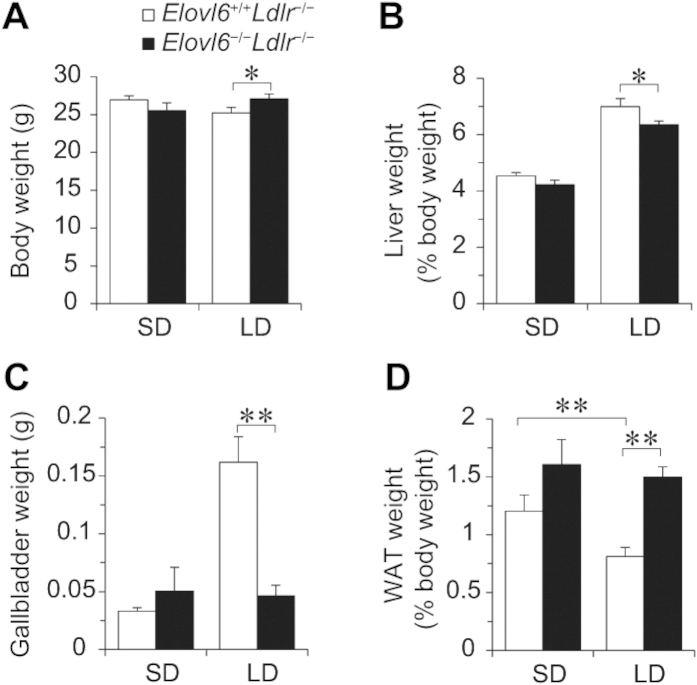
Total body, liver, white adipose tissue (WAT) and gallbladder weights of *Ldlr*^−/−^ mice lacking Elovl6. (**A**) Whole body, (**B**) liver, (**C**) gallbladder and (**D**) perigonadal WAT weights were measured in *Elovl6*^+/+^*Ldlr*^−/−^ and *Elovl6*^−/−^*Ldlr*^−/−^ mice fed a standard diet (SD) or a lithogenic diet (LD) for 4 weeks (n = 8–13 per group, ^*^P < 0.05, ^**^P < 0.01).

Elovl6 and LDLR deficient mice were crossed to generate mice with deficiencies of both Elovl6 (*Elovl6*^−/−^) and LDLR (*Ldlr*^−/−^). Mice aged 11–16 weeks were fed a standard diet (SD) or an LD for 4 weeks. On an SD, *Elovl6*^−/−^*Ldlr*^−/−^ mice had similar body weight to the *Elovl6*^+/+^*Ldlr*^−/−^ mice (Fig. 1A). The LD showed a trend to decrease body weight in *Elovl6*^+/+^*Ldlr*^−/−^ mice and to increase body weight in *Elovl6*^−/−^*Ldlr*^−/−^ mice. As a result, LD-fed *Elovl6*^+/+^*Ldlr*^−/−^ mice were significantly lighter than LD-fed *Elovl6*^−/−^*Ldlr*^−/−^ mice. Daily food intake in LD-fed *Elovl6*^−/−^*Ldlr*^−/−^ mice was not significantly different compared with LD-fed *Elovl6*^+/+^*Ldlr*^−/−^ mice. The LD for 4 weeks increased liver weights in both *Elovl6*^+/+^*Ldlr*^−/−^ and *Elovl6*^−/−^*Ldlr*^−/−^ mice compared with their SD-fed counterparts, but *Elovl6*^−/−^*Ldlr*^−/−^ mice showed less LD-induced liver weight gain compared with *Elovl6*^+/+^*Ldlr*^−/−^ mice (Fig. 1B). It is well known that an LD increases the amount of bile, leads to supersaturation of the bile with cholesterol and increases susceptibility to gallstone formation. Supporting this, gallbladder weights were markedly increased in LD-fed *Elovl6*^+/+^*Ldlr*^−/−^ mice (Fig. 1C). Conversely, absence of Elovl6 abolished LD-induced gallbladder enlargement, indicating a decrease in the amount of bile in *Elovl6*^−/−^*Ldlr*^−/−^ mice. LD-fed *Elovl6*^+/+^*Ldlr*^−/−^ mice exhibited significant reductions in epididymal white adipose tissue (WAT) weight compared with SD-fed *Elovl6*^+/+^*Ldlr*^−/−^ mice (Fig. 1D). In contrast, there was no significant difference in WAT weight between LD-fed and SD-fed *Elovl6*^−/−^*Ldlr*^−/−^ mice.

### Elovl6 deficiency reduces plasma total cholesterol and bile acid levels in LD-fed *Ldlr* ^**−**/**−**^ mice

**Figure 2:**
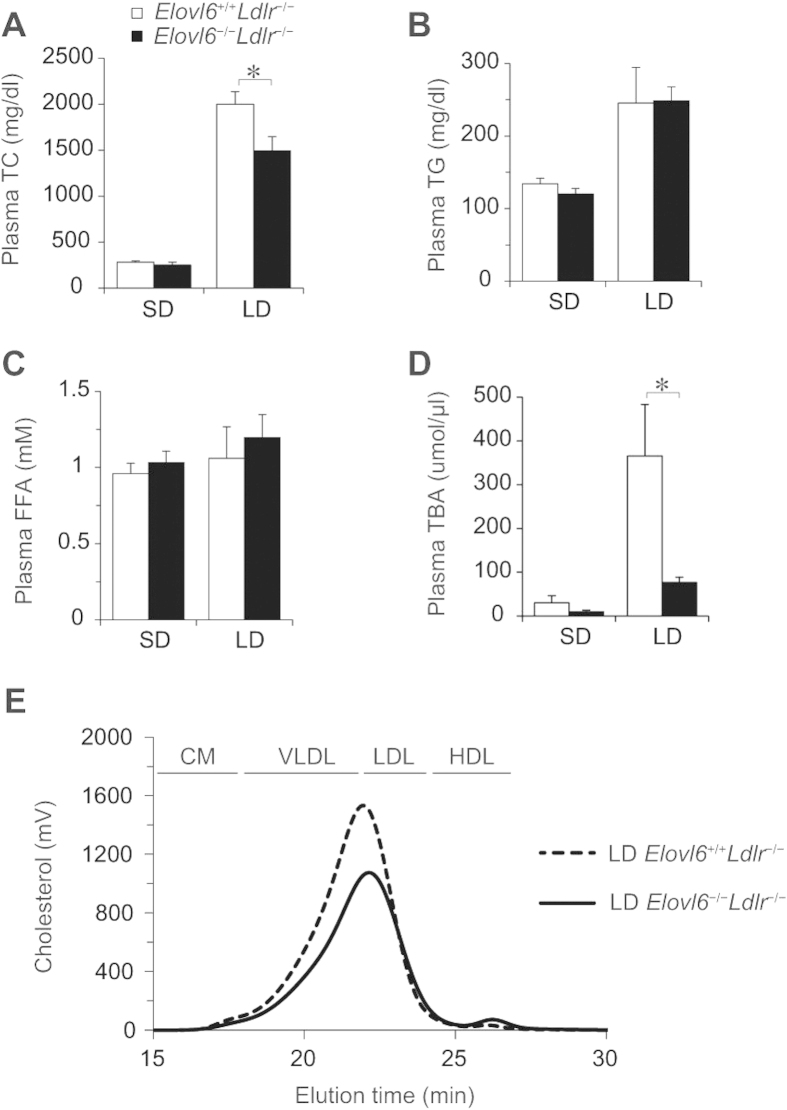
Plasma lipid and lipoprotein profiles of lithogenic diet (LD)-fed *Ldlr*^−/−^ mice lacking Elovl6. (**A**) Plasma total cholesterol (TC), (**B**) triglyceride (TG), (**C**) free fatty acid (FFA) and (**D**) total bile acid (TBA) levels in *Elovl6*^+/+^*Ldlr*^−/−^ and *Elovl6*^−/−^*Ldlr*^−/−^ mice fed a standard diet (SD) or an LD for 4 weeks (n = 8–13 per group). (**E**) High performance liquid chromatography lipoprotein profiles of pooled plasma samples from *Elovl6*^+/+^*Ldlr*^−/−^ and *Elovl6*^−/−^*Ldlr*^−/−^ mice fed an LD for 4 weeks (n = 3–4 per group). Peaks for very low density lipoprotein (VLDL), low density lipoprotein (LDL) and high density lipoprotein (HDL) are indicated. ^*^P < 0.05, ^**^P < 0.01.

In mice fed an SD, plasma levels of total cholesterol (TC), TG, free fatty acid (FFA) and total bile acid (TBA) were not significantly different between *Elovl6*^+/+^*Ldlr*^−/−^ and *Elovl6*^−/−^*Ldlr*^−/−^ mice (Fig. 2A–D). After 4 weeks on an LD, plasma TC, TG and TBA levels in *Elovl6*^+/+^*Ldlr*^−/−^ mice were markedly elevated compared with levels in SD-fed *Elovl6*^+/+^*Ldlr*^−/−^ mice as a consequence of dietary loading of cholesterol, fat and cholic acid (CA) in the LD. However, *Elovl6*^−/−^*Ldlr*^−/−^ mice were strongly resistant to these changes caused by the LD. Compared with LD-fed *Elovl6*^+/+^*Ldlr*^−/−^ mice, LD-fed *Elovl6*^−/−^*Ldlr*^−/−^ mice had significantly lower plasma TC, and plasma TBA levels were markedly suppressed (Fig. 2A, D). High-performance liquid chromatography (HPLC) revealed that very low density lipoprotein (VLDL) and LDL cholesterol, the major cholesterol fraction in *Ldlr*^−/−^ mice on an LD, was reduced and HDL cholesterol was increased in LD-fed *Elovl6*^−/−^*Ldlr*^−/−^ mice compared with LD-fed *Elovl6*^+/+^*Ldlr*^−/−^ mice (Fig. 2E).

### Elovl6 deficiency suppresses hepatic lipid accumulation but promotes cholesterol crystallization and gallstone formation in *Ldlr* ^**−**/**−**^ mice

**Figure 3:**
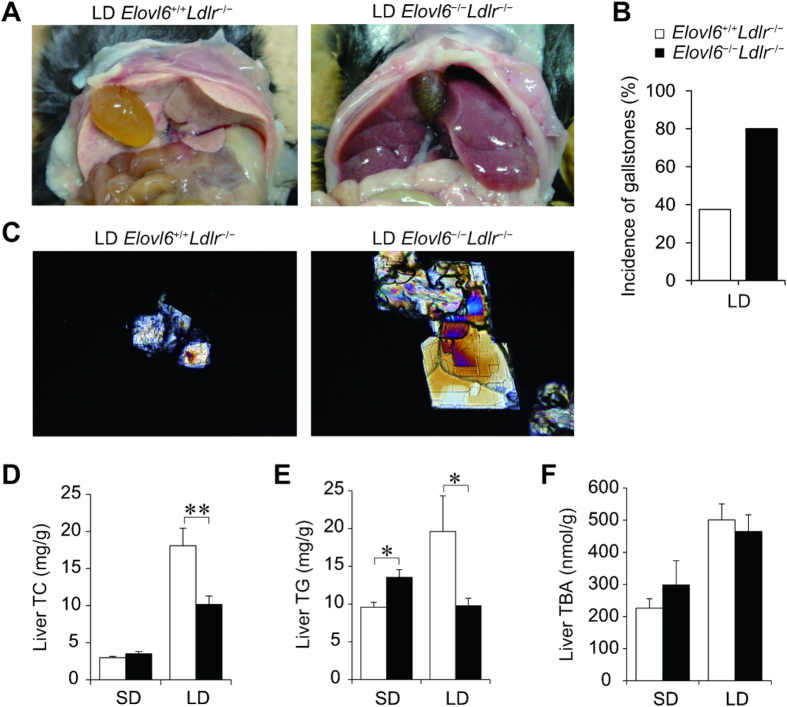
Elovl6 deficiency suppresses lithogenic diet (LD)-induced hepatic lipid accumulation in *Ldlr*^−/−^ mice. (**A**) Representative photographs of livers from *Elovl6*^+/+^*Ldlr*^−/−^ and *Elovl6*^−/−^*Ldlr*^−/−^ mice fed an LD for 4 weeks. (**B**) The prevalence rate of gallstones in *Elovl6*^+/+^*Ldlr*^−/−^ and *Elovl6*^−/−^*Ldlr*^−/−^ mice fed an LD for 4 weeks (n = 8–10 per group). (**C**) Polarizing light microscopy of gallbladder bile from *Elovl6*^+/+^*Ldlr*^−/−^ and *Elovl6*^−/−^*Ldlr*^−/−^ mice fed an LD for 4 weeks (magnification, ×200). (**D–F**) Hepatic total cholesterol (TC) (**D**), triglyceride (TG) (**E**) and total bile acid (TBA) (**F**) levels in *Elovl6*^+/+^*Ldlr*^−/−^ and *Elovl6*^−/−^*Ldlr*^−/−^ mice fed a standard diet (SD) or an LD for 4 weeks (n = 8–13 per group). *P < 0.05, **P < 0.01.

Photographs of the opened abdominal cavities of both groups on the LD show that the livers of LD-fed *Elovl6*^+/+^*Ldlr*^−/−^ mice were pale, but the livers of LD-fed *Elovl6*^−/−^*Ldlr*^−/−^ mice were red (Fig. 3A). However, in contrast to *Elovl6*^+/+^*Ldlr*^−/−^ mice after 4 weeks of the LD, the LD-fed *Elovl6*^−/−^*Ldlr*^−/−^ mice had opaque gallbladders and increased numbers of aggregated cholesterol gallstones in greenish bile. The incidence of gallstones was higher in *Elovl6*^−/−^*Ldlr*^−/−^ mice (80%) than in *Elovl6*^+/+^*Ldlr*^−/−^ mice (37.5%) after 4-week feeding of LD (Fig. 3B). Microscopic examination of the gallbladder bile from LD-fed *Elovl6*^−/−^*Ldlr*^−/−^ mice revealed numerous large cholesterol monohydrate crystals; whereas, bile from LD-fed *Elovl6*^+/+^*Ldlr*^−/−^ mice was largely free of cholesterol precipitates, with only occasional aggregated vesicles in most animals (Fig. 3C). The LD markedly increased liver TC and increased liver TG levels in *Elovl6*^+/+^*Ldlr*^−/−^ mice. In LD-fed *Elovl6*^−/−^*Ldlr*^−/−^ mice, the liver TC increase was reduced to half that observed in *Elovl6*^+/+^*Ldlr*^−/−^ mice and there was no increase in TG levels (Fig. 3D, E). The LD increased liver TBA levels in both genotypes, but unlike plasma levels, there was no significant difference between the groups (Fig. 3F). Bile TC, TBA, phospholipid (PL), faecal TC and TBA levels were similar between LD-fed *Elovl6*^+/+^*Ldlr*^−/−^ and LD-fed *Elovl6*^−/−^*Ldlr*^−/−^ mice (see Supplementary Fig. S1 online). These results suggest that it was the change in hepatic lipid metabolism in LD-fed *Elovl6*^*−/−*^*Ldlr*^*−/−*^ mice that suppressed hepatic cholesterol and triglycerides accumulation and enhanced gallstone formation, rather than a functional change in the gallbladder or intestine.

**Figure 4:**
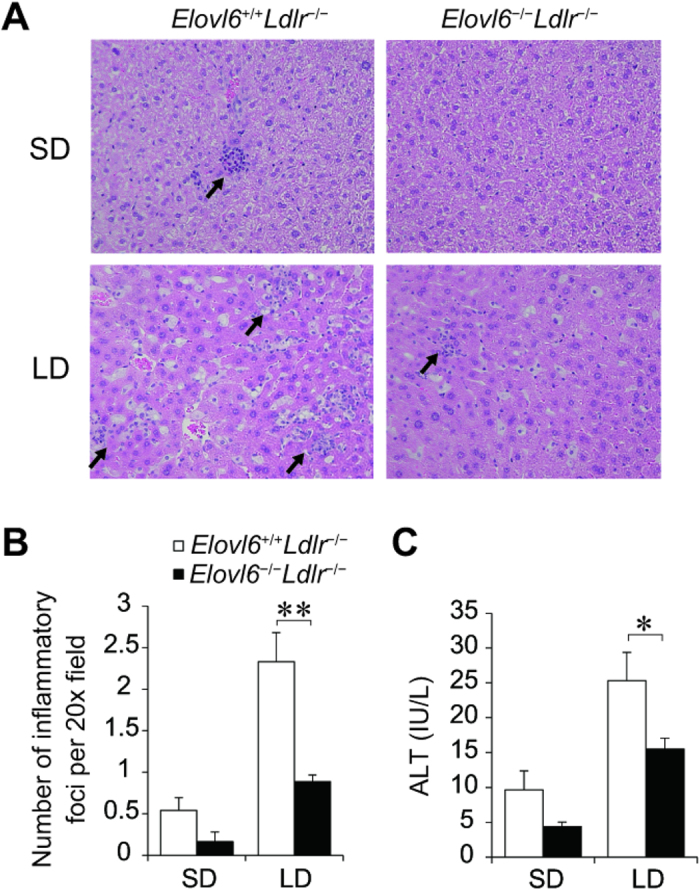
Attenuated hepatic inflammation and liver injury in lithogenic diet (LD)-fed *Elovl6*^−/−^*Ldlr*^−/−^ mice. (**A**) Representative hematoxylin and eosin (H&E)-stained sections of livers from *Elovl6*^+/+^*Ldlr*^−/−^ and *Elovl6*^−/−^*Ldlr*^−/−^ mice fed a standard diet (SD) or an LD for 4 weeks. (**B**) The number of inflammatory foci in H&E-stained sections from each animal at 20× magnification (n = 6–9 per group). (**C**) Plasma alanine aminotransferase (ALT) levels of *Elovl6*^+/+^*Ldlr*^−/−^ and *Elovl6*^−/−^*Ldlr*^−/−^ mice fed an SD or an LD for 4 weeks (n = 8–13 per group). ^*^P < 0.05, ^**^P < 0.01.

Histological examination of the livers revealed some inflammatory cell infiltration in SD-fed *Elovl6*^+/+^*Ldlr*^−/−^ mice, but not in SD-fed *Elovl6*^−/−^*Ldlr*^−/−^mice (Fig. 4A). Inflammatory cell infiltration was exacerbated in LD-fed *Elovl6*^+/+^*Ldlr*^−/−^ mice, but markedly ameliorated in LD-fed *Elovl6*^−/−^*Ldlr*^−/−^ mice. The protection from inflammation and liver damage associated with Elovl6 deficiency was reflected in a significantly lower hepatic lobular inflammatory grade (evaluated by the number of inflammatory foci in hematoxylin & eosin [H&E]-stained liver sections) and plasma alanine aminotransferase (ALT) levels in LD-fed *Elovl6*^−/−^*Ldlr*^−/−^ mice compared with LD-fed *Elovl6*^+/+^*Ldlr*^−/−^ mice (Fig. 4B,C).

### Elovl6 deficiency leads to decreased hepatic C18:1 in LD-fed *Ldlr* ^**−**/**−**^ mice

**Figure 5:**
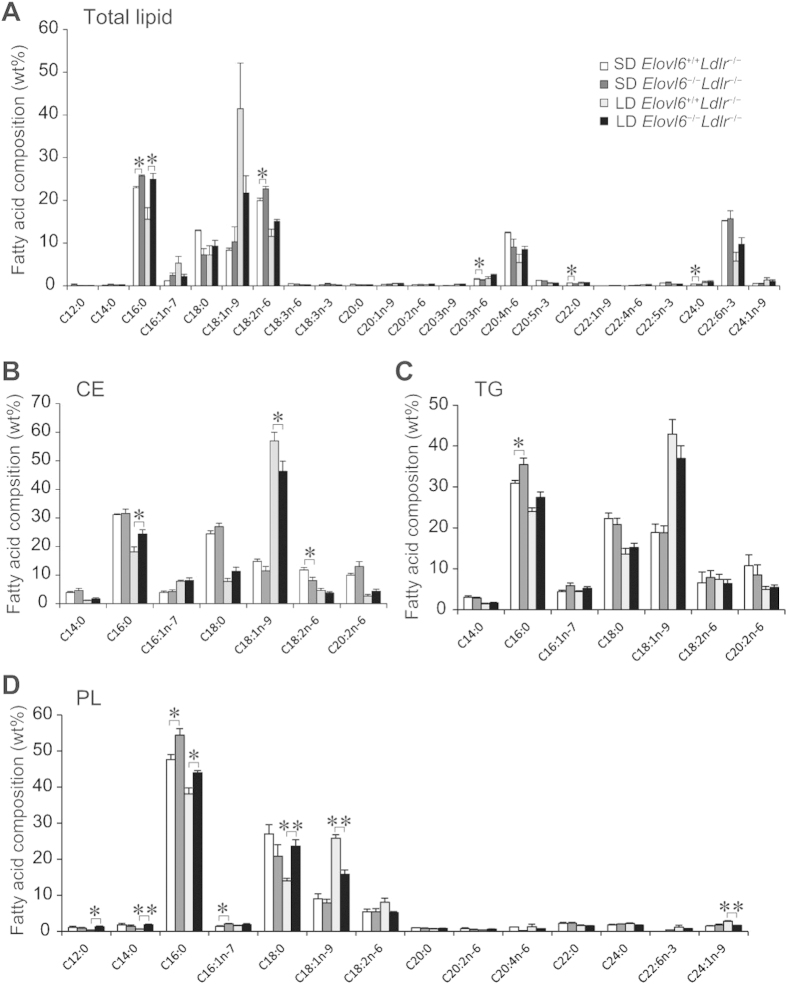
Hepatic fatty acid composition. (**A**) Hepatic fatty acid composition for total lipids, **(B)** the cholesterol ester (CE) fraction, **(C)** the triglyceride (TG) fraction and **(D)** the phospholipid (PL) fraction in livers from *Elovl6*^+/+^*Ldlr*^−/−^ and *Elovl6*^−/−^*Ldlr*^−/−^ mice fed a standard diet (SD) or a lithogenic diet (LD) for 4 weeks. Hepatic total lipids were extracted, and the major classes of lipids were separated on a silica column. The lipid fractions were methyl-esterified and quantified by gas chromatography (n = 3–6 per group). ^*^P < 0.05, ^**^P < 0.01.

FA composition analysis identified that lack of Elovl6 modified the FA profiles of total lipid, cholesterol ester (CE), TG and PL fractions in the livers of *Ldlr*^−/−^ mice fed an SD or an LD (Fig. 5). Compared with SD-fed *Elovl6*^+/+^*Ldlr*^−/−^ mice, the total lipid fractions of SD-fed *Elovl6*^−/−^*Ldlr*^−/−^ mice had increased C16:0 and C18:2n-6 and decreased C20:3n-6, C22:0 and C24:0 (Fig. 5A). Compared with LD-fed *Elovl6*^+/+^*Ldlr*^−/−^ mice, the total lipid fractions of LD-fed *Elovl6*^−/−^*Ldlr*^−/−^ mice had increased C16:0 and a tendency toward decreased C18:1n-9. In the CE fraction, compared with LD-fed *Elovl6*^+/+^*Ldlr*^−/−^ mice, the livers of the LD-fed *Elovl6*^−/−^*Ldlr*^−/−^ mice had a decreased relative amount of C18:1n-9, whereas the relative amount of C16:0 was increased (Fig. 5B). In the TG fraction, the only significant difference between the groups was increased C16:0 in the livers of SD-fed *Elovl6*^−/−^*Ldlr*^−/−^ mice compared with SD-fed *Elovl6*^+/+^*Ldlr*^−/−^ mice (Fig. 5C). In the PL fraction, the relative amounts of C16:0 and C16:1n-7 were increased in the livers of SD-fed *Elovl6*^−/−^*Ldlr*^−/−^ mice compared with SD-fed *Elovl6*^+/+^*Ldlr*^−/−^ mice (Fig. 5D). In the PL fraction in LD-fed mice, the relative amounts of C12:0, C14:0, C16:0, C16:1n-7, and C18:0 were increased and the relative amounts of C18:1n-9 and C24:1n-9 were decreased in the livers of *Elovl6*^−/−^*Ldlr*^−/−^ mice compared with *Elovl6*^+/+^*Ldlr*^−/−^ mice. Despite different degrees of change depending on the lipid fraction, the overall changes in FA composition in both genotypes were consistent with Elovl6 enzymatic activity.

### Elovl6 deficiency alters bile acid composition in the livers and bile of *Ldlr* ^**−**/**−**^ mice

The composition of BAs is associated with hepatotoxicity and cholesterol crystallization^24^,^25^,^26^,^27^. To investigate a potential role of Elovl6 on BA metabolism and whether BA alterations could contribute to reduced LD-induced liver damage and gallstone formation in LD-fed *Elovl6*^−/−^*Ldlr*^−/−^ mice, the composition of individual BAs in the livers of *Elovl6*^*+/+*^*Ldlr*^*−/−*^ and *Elovl6*^−/−^*Ldlr*^−/−^ mice fed an SD or an LD were quantified by liquid chromatography/mass spectrometry (LC/MS) (see Supplementary Fig. S2 online). Determination of individual BA species revealed that taurocholic acid (TCA), tauro-β-muricholate (TβMCA) and tauro-ω-muricholate (TωMCA) represent the dominant BA species in SD-fed animals of both genotypes. The ablation of Elovl6 in *Ldlr*^−/−^ mice had little effect on liver BA composition, except that the ratio of tauro-α-muricholate (TαMCA) was significantly decreased in the livers of *Elovl6*^−/−^*Ldlr*^−/−^ mice compared with *Elovl6*^+/+^*Ldlr*^−/−^ mice. Because of CA-overloading on the LD, there was a shift in the liver BA species in response to LD-feeding, with CA and its derivatives, TCA and taurodeoxycholic acid (TDCA), representing the major species in both genotypes. Elovl6 deficiency significantly decreased the ratio of TDCA and TαMCA and significantly increased the ratio of glycocholic acid (GCA) in the livers of LD-fed *Ldlr*^−/−^ mice. TDCA is increased in NASH patients^26^, providing a plausible biochemical explanation for the LD phenotype of *Elovl6*^−/−^*Ldlr*^−/−^ mice. Similar BA compositional changes were seen in bile (see Supplementary Fig. S2 online). In contrast, lack of Elovl6 in *Ldlr*^−/−^ mice had little effect on BA composition in plasma despite strong suppression of the total BA level. However, lack of Elovl6 in *Ldlr*^−/−^ mice was associated with an increased concentration of chenodeoxycholic acid (CDCA) in an LD-fed state (see Supplementary Fig. S2 online).

### Elovl6 deficiency alters LD-responsive gene expression in the livers of *Ldlr* ^**−**/**−**^ mice

**Figure 6:**
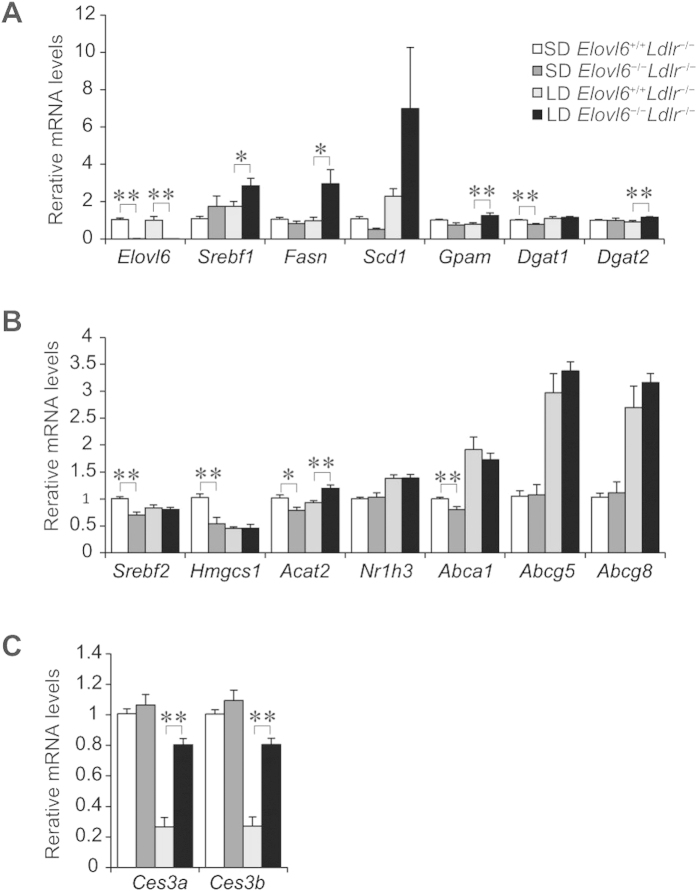
Quantitative real-time PCR (qPCR) analysis of genes involved in steatohepatitis. *Elovl6*^+/+^*Ldlr*^−/−^ and *Elovl6*^−/−^*Ldlr*^−/−^ mice were fed a standard diet (SD) or a lithogenic diet (LD) for 4 weeks and sacrificed following 4 h of food deprivation (n = 8–13 per group). (**A**) qPCR analysis of genes for fatty acid and triglyceride synthesis, (**B**) cholesterol metabolism and (**C**) carboxylesterase. ^*^P < 0.05, ^**^P < 0.01.

To have some idea on the molecular mechanisms by which an LD results in such a striking phenotype in *Elovl6*^−/−^*Ldlr*^−/−^ mice, mRNA expression of candidate genes in livers from mice of both genotypes fed an SD or an LD diet was examined using quantitative real-time polymerase chain reaction (qPCR). Genes involved in FA synthesis controlled by sterol regulatory element binding protein 1c (*Srebf1c*), such as fatty acid synthase (*Fasn*), were significantly increased and stearoyl-CoA desaturase-1 (*Scd1*) showed a tendency to increase in the livers of LD-fed *Elovl6*^−/−^*Ldlr*^−/−^ mice compared with LD-fed *Elovl6*^+/+^*Ldlr*^−/−^ mice (Fig. 6A). Expression of genes involved in TG synthesis, including glycerol-3-phosphate acyltransferase (*Gpam*) and diacylglycerol O-acyltransferase 2 (*Dgat2*), was slightly increased in the livers of LD-fed *Elovl6*^−/−^*Ldlr*^−/−^ mice compared with LD-fed *Elovl6*^+/+^*Ldlr*^−/−^ mice; whereas, expression of diacylglycerol O-acyltransferase 1 (*Dgat1*) was similar in both genotypes. Expression of genes involved in cholesterol synthesis regulated by sterol regulatory element binding protein 2 (*Srebp2*), including 3-hydroxy-3-trmethylglutaryl-coenzyme A synthase 1 (*Hmgcs1*), was decreased, and expression of genes for cholesterol efflux enhanced by liver X receptor α (Lxrα) (nuclear receptor subfamily 1, group H, member 3: *Nr1h3*), such as ATP-binding cassette sub-family A member 1 (*Abca1*), ATP-binding cassette sub-family G member 1 (*Abcg5*), and *Abcg8*, was increased in response to cholesterol feeding in both groups of mice (Fig. 6B). Expression of acetyl-coenzyme A acetyltransferase 2 (*Acat2*) was increased in LD-fed *Elovl6*^−/−^*Ldlr*^−/−^ mice compared with LD-fed *Elovl6*^+/+^*Ldlr*^−/−^ mice. The enhanced expression of lipogenic enzymes and *Acat2* in the livers of *Elovl6*^−/−^*Ldlr*^−/−^ mice might compensate for the unbalanced FA composition. Expression of carboxylesterase 3a and 3b (*Ces3a* and *Ces3b*) was suppressed by the LD in *Elovl6*^+/+^*Ldlr*^−/−^ mice, but was restored by the absence of Elovl6 (Fig. 6C).

**Figure 7:**
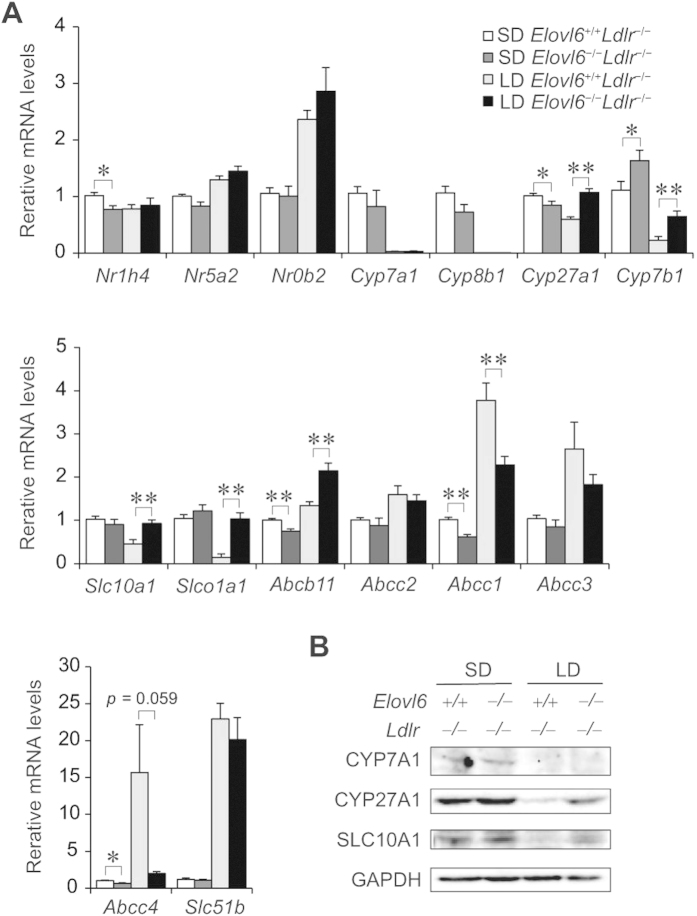
Hepatic expression levels of genes involved in BA metabolism. *Elovl6*^+/+^*Ldlr*^−/−^ and *Elovl6*^−/−^*Ldlr*^−/−^ mice were fed a standard diet (SD) or a lithogenic diet (LD) for 4 weeks and sacrificed following 4 h of food deprivation (n = 8–13 per group). (**A**) qPCR analysis of genes for BA metabolism. ^*^P < 0.05, ^**^P < 0.01. (**B**) Immunoblot analysis of CYP7A1, CYP27A1, SLC10A1 and glyceraldehyde 3-phosphate dehydrogenase (GAPDH) in the livers of *Elovl6*^+/+^*Ldlr*^−/−^ and *Elovl6*^−/−^*Ldlr*^−/−^ mice fed a SD or a LD for 4 weeks.

The expression of genes associated with BA metabolism was also examined (Fig. 7A). Loss of Elovl6 slightly decreased the expression of farnesoid X receptor (Fxr) (nuclear receptor subfamily 1, group H, member 4: *Nr1h4*) in mice on the SD but had little effect in the LD-fed mice. The LD increased the expression of the Fxr-target genes, liver receptor homolog-1 (Lrh1) (nuclear receptor subfamily 5, group A, member 2: *Nr5a2*) and small heterodimer partner (Shp) (nuclear receptor subfamily 0, group B, member 2: *Nr0b2*), in both genotypes. The biosynthesis of BAs in the liver is controlled by multiple cytochrome P450 (CYP) enzymes^28^. The LD markedly suppressed mRNA expression of cholesterol 7α-hydroxylase (*Cyp7a1*) and sterol 12α-hydroxylase (*Cyp8b1*), the two key enzymes in BA synthesis, in both genotypes. Expression of sterol 27-hydroxylase (*Cyp27a1*) and oxysterol 7α-hydroxylase (*Cyp7b1*), which are involved in an alternative pathway of BA synthesis, was also decreased by the LD in *Elovl6*^+/+^*Ldlr*^−/−^ mice, but was significantly upregulated in the livers of LD-fed *Elovl6*^−/−^*Ldlr*^−/−^ mice compared with LD-fed *Elovl6*^+/+^*Ldlr*^−/−^ mice. Expression of solute carrier family 10 member 1 (*Slc10a1*) and solute carrier organic anion transporter family member 1a1 (*Slco1a1*), which are involved in BA uptake by hepatocytes, was suppressed by the LD in *Elovl6*^+/+^*Ldlr*^−/−^ mice. Strikingly, LD-fed *Elovl6*^−/−^*Ldlr*^−/−^ mice displayed a complete lack of *Slc10a1* and *Slco1a1* suppression. Expression of ATP-binding cassette sub-family B member 11 (*Abcb11*), an ABC transporter responsible for the transport of bile salt from hepatocytes into the bile, was significantly upregulated in the livers by the LD with greater intensity in *Elovl6*^−/−^*Ldlr*^−/−^ mice compared to LD *Elovl6*^+/+^*Ldlr*^−/−^ mice. Expression of ATP-binding cassette subfamily C member 1 (*Abcc1*), *Abcc3 and Abcc4*, which are involved in basolateral BA excretion, was markedly elevated in LD-fed *Elovl6*^+/+^*Ldlr*^−/−^ mice. The expression of *Abcc1* was significantly decreased, and *Abcc4* tended to decrease (*P* = 0.059) in LD-fed *Elovl6*^−/−^*Ldlr*^−/−^ mice compared with LD-fed *Elovl6*^+/+^*Ldlr*^−/−^ mice. Consistently with mRNA levels, hepatic CYP7A1 protein was suppressed by LD feeding in both genotypes (Fig. 7B). Hepatic protein levels of CYP27a1 and SLC10A1 was also decreased by the LD in *Elovl6*^+/+^*Ldlr*^−/−^ mice, but was upregulated in LD-fed *Elovl6*^−/−^*Ldlr*^−/−^ mice compared with LD-fed *Elovl6*^+/+^*Ldlr*^−/−^ mice.

The expression of genes involved in BA homeostasis in ileum was also examined (Supplementary Fig. S3). The expression levels of *Nr1h4*, solute carrier family 10 member 2 (*Slc10a2*), *Abcc2*, fatty acid binding protein 6 ileal (*Fabp6*), solute carrier family 51 beta subunit (*Slc51b*) and fibroblast growth factor 15 (*Fgf15*) were similar between LD-fed *Elovl6*^+/+^*Ldlr*^−/−^ and LD-fed *Elovl6*^−/−^*Ldlr*^−/−^ mice.

### Elovl6 deletion attenuates the inflammatory response and hepatic injury in LD-fed *Ldlr* ^**−**/**−**^ mice

**Figure 8:**
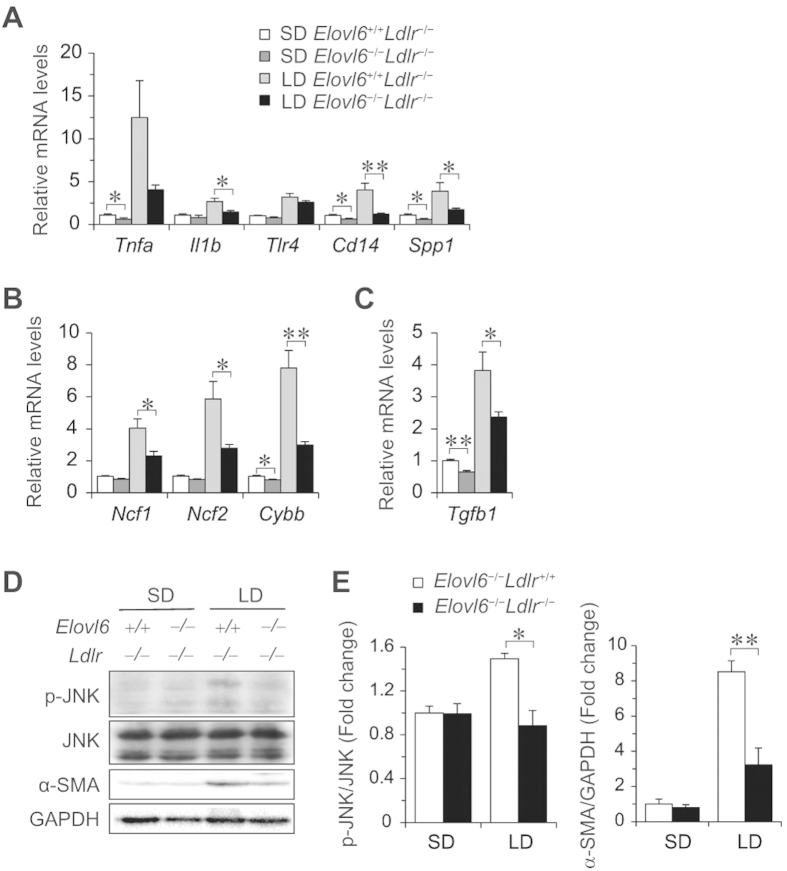
Effects of Elovl6 deficiency on hepatic mRNA and levels of proteins involved in inflammation, oxidative stress and fibrosis. (**A–C**) Quantitative real-time PCR (qPCR) analysis of genes for inflammation (**A**), reactive oxygen species (ROS) generation (**B**) and fibrogenesis (**C**). (**D**) Immunoblot analysis of phosphorylated and total anti-phospho-c-Jun N-terminal kinase (JNK), α-smooth muscle actin (α-SMA) and GAPDH in the livers of *Elovl6*^+/+^*Ldlr*^−/−^ and *Elovl6*^−/−^*Ldlr*^−/−^ mice fed a standard diet (SD) or a lithogenic diet (LD) for 4 weeks. (**E**) Ratio between phosphorylated and total JNK and α-SMA and GAPDH on densitometry analysis (n = 2–4 per group). ^*^P < 0.05, ^**^P < 0.01.

Hepatic damage in LD-fed *Ldlr*^−/−^ mice is associated with hepatic inflammation, oxidative stress and fibrosis. In agreement with histological and metabolic observations (Fig. 4), inflammatory response genes including tumour necrosis factor α (*Tnfα*), interleukin 1 β (*Il-1b*), toll-like receptor 4 (*Tlr4*), CD14 antigen (*Cd14*) and secreted phosphoprotein 1 (*Spp1*) were upregulated in the livers of LD-fed *Elovl6*^+/+^*Ldlr*^−/−^ mice (Fig. 8A). However, induction of these genes showed marked or a trend to suppression in LD-fed *Elovl6*^−/−^*Ldlr*^−/−^ mice. The expression levels of genes for the nicotinamide adenine dinucleotide phosphate (NADPH) oxidase complex, neutrophil cytosolic factor 1 (*Ncf1*), neutrophil cytosolic factor 2 (*Ncf2*) and cytochrome b-245 beta polypeptide (*Cybb*), were upregulated in LD-fed *Elovl6*^+/+^*Ldlr*^−/−^ mice, but significantly decreased in LD-fed *Elovl6*^−/−^*Ldlr*^−/−^ mice compared with LD-fed *Elovl6*^+/+^*Ldlr*^−/−^ mice (Fig. 8B). The expression level of the gene for transforming growth factor β 1 (*Tgfb1*) was upregulated in LD-fed *Elovl6*^+/+^*Ldlr*^−/−^ mice but suppressed in LD-fed *Elovl6*^−/−^*Ldlr*^−/−^ mice compared with LD-fed *Elovl6*^+/+^*Ldlr*^−/−^ mice (Fig. 8C). Decreased hepatic inflammation, oxidative stress and fibrosis in *Elovl6*^−/−^*Ldlr*^−/−^ mice were further examined by measuring stresses and proinflammatory pathways. Elovl6 deficiency significantly attenuated LD-induced c-Jun N-terminal kinase (JNK) activation and α-smooth muscle actin (α-SMA) levels in *Ldlr*^−/−^ mice (Fig. 8D,E).

## Discussion

Our current findings show that the absence of the *Elovl6* gene product strongly inhibits LD-induced accumulation of hepatic and plasma cholesterol and plasma TBA in *Ldlr*^−/−^ mice. The absence of Elovl6 also imparts striking protection from LD-induced hepatic injury by ameliorating hepatic inflammation, oxidative stress and stellate cell activation. The net results of these complex adaptations are a striking decrease in susceptibility to NASH, but increased susceptibility to gallstones. Normolipidemic Elovl6-deficient mice are known to be protected from AHF-induced NASH with no change in hepatic lipid content or profiles^23^. We have now extended the findings in *Elovl6*^−/−^ mice to include protection from diet-induced hepatosteatosis and inflammation in dyslipidemic LDLR-deficient mice.

Feeding an LD (cholesterol and CA loading) causes accumulation of plasma, hepatic and bile cholesterols in *Ldlr*^−/−^ mice in response to activation of both LXR and FXR pathways and suppression of SREBP-2. Plasma and hepatic BAs were also elevated despite adaptive responses to hepatic handling of BA, such as decreased hepatic uptake, increased basolateral excretion and enhanced excretion into bile, as evidenced by changes in gene expression of BA transporters and pumps to prevent overaccumulation of BA in hepatocytes (as schematized in Supplementary Fig. S4). Meanwhile, the absence of Elovl6 ameliorated LD-induced accumulation of lipids and BA in *Ldlr*^−/−^ mice, indicating that Elovl6 and FA composition play a role in cholesterol and BA handling in hepatocytes.

A potential mechanism for the reduction in hepatic and plasma cholesterol in LD-fed *Elovl6*^−/−^*Ldlr*^−/−^ mice is the suppression of esterification of cholesterol secondary to decreased oleate (C18:1) levels in the liver. Elovl6 deficiency significantly reduced oleate levels in CE and PL fractions. Because oleate, the major esterified cholesterol in liver, is a better substrate for ACAT than palmitate, inhibition of the conversion from palmitate to stearate resulting in decreased endogenous monounsaturated FAs could lead to reduction in esterified cholesterol. Expression of *Ces3a* and *Ces3b* was strongly suppressed by the LD in *Elovl6*^+/+^*Ldlr*^−/−^ mice and restored in *Elovl6*^−/−^*Ldlr*^−/−^ mice. Esterase and lipase activities of these genes might contribute to decreased hepatic lipid content in LD-fed *Elovl6*^−/−^*Ldlr*^−/−^ mice. Our data are consistent with previous studies showing that mice harbouring a natural mutation in the *Scd1* gene are deficient in hepatic CE and TG with decreased monounsaturated FAs (C16:1 and C18:1), and indicate that a reduction in hepatic oleate is likely to be a major contributor to decreased hepatic and plasma cholesterol^29^,^30^. It has also been reported that monounsaturated FA-containing CE levels were markedly elevated in the livers of mice fed a cholate-supplemented high-fat diet, but without an increase in the level of TG or PL in the liver, coupled with the biological and histological features of fatty liver injury^31^. Moreover, increased oleate levels have been found in the livers of NASH patients^32^. Recent publications have also indicated that hepatic cholesterol accumulation is more toxic than TG accumulation and may be a trigger for the progression of NAFLD^13^,^33^,^34^. Therefore, these data indicate that the excessive accumulation of CE with oleate might contribute to liver injury and that consequent loss of oleate resulting from the absence of *Elovl6* could be the cause of the amelioration in the current study.

Our data are consistent with our recent study using an AHF diet-fed mouse model of NAFLD/NASH, which demonstrated a critical role for Elovl6 in disease pathogenesis^23^. In that study, *Elovl6*^−/−^ mice displayed a significant decrease in hepatic inflammation, oxidative stress, liver injury and fibrosis, but without amelioration of hepatosteatosis. The finding of protection against steatosis in the present study, which is in contrast to the previous study, is presumably because the current study protocol highlights cholesterol rather than TG accumulation in hepatosteatosis. LDLR deficiency activates cholesterol synthesis in the liver. In addition, the LD has a lower FA content than the AHF diet. Therefore, in an LD-fed *Ldlr*^−/−^ mouse model, the reduction of oleate by Elovl6 deficiency is further reflected in CE formation. Our previous study also showed a reduction in esterified cholesterol accumulation in macrophages from *Elovl6*^−/−^ mice^18^.

One of the intriguing findings of the present study was the protection against LD-induced serum BA elevation coupled with altered expression of genes involved in hepatic BA metabolism (*Cyp27a1*, *Cyp7b1*, *Slc10a1*, *Slco1a1*, *Abcb11* and *Abcc1*) in LD-fed *Elovl6*^−/−^*Ldlr*^−/−^ mice. *Cyp27a1* and *Cyp7b1* expression were significantly increased in the livers of *Elovl6*^−/−^*Ldlr*^−/−^ mice compared with *Elovl6*^*+*/+^*Ldlr*^−/−^ mice fed on both an SD and an LD. The alternative pathway may contribute very little to overall BA synthesis under normal conditions, but may become more predominant in the presence of liver disease and may compensate for the limitations in the classical pathway^35^,^36^. Increased *Cyp27a1* and *Cyp7b1* mRNA levels are indicative of a functional upregulation of the alternative pathway to reduce hepatotoxicity and the progression of liver disease to NASH^37^. In addition, *Cyp7b1* expression was negatively associated with hepatic steatosis in an animal model^38^. Therefore, the increase in hepatic *Cyp7b1* expression might contribute to the suppression of hepatosteatosis and liver injury in LD-fed *Elovl6*^−/−^*Ldlr*^−/−^ mice. In the present study, the expression of *Slc10a1* and *Slco1a1* was increased in LD-fed *Elovl6*^−/−^*Ldlr*^−/−^ mice in concert with decreased expression of *Abcc1* and *Abcc4*. In support of these observations, increases in serum BAs were related to a decrease and an increase in *Slco1a1* and *Abcc4* expression, respectively, in the livers of mouse NASH models^39^. These data suggest that LD-fed *Elovl6*^−/−^*Ldlr*^−/−^ mice have a greater ability to remove BAs from the hepatic-portal circulation than LD-fed *Elovl6*^+/+^*Ldlr*^−/−^ mice.

In the present study, the LD significantly decreased the expression of *Slc10a and Slco1a1* and increased the expression of *Abcc1 and Abcc4* in the livers of *Elovl6*^+/+^*Ldlr*^−/−^ mice, whereas the expression of these genes remained unchanged in LD-fed *Elovl6*^−/−^*Ldlr*^−/−^ mice. It is well known that inflammatory signals act as potent regulators of the expression of sinusoidal and basolateral BA transporters. For example, lipopolysaccharide (LPS) downregulates the expression of *Slc10a1* and *Slco1a1* and increases the expression of *Abcc1*^40^. In addition, depletion of Kupffer cells inhibits the LPS-induced downregulation of *Slco1a1* and upregulation of *Abcc4* by attenuating the increase in TNF-α expression^41^,^42^. These previous observations support the present results and demonstrate one of the mechanisms by which inflammatory signals disrupt BA homeostasis in the liver. Because Elovl6 deficiency or modification of FA composition causes a drastic change in cholesterol metabolism, Elovl6 might also regulate BA handling (hepatic production, uptake, basolateral and canalicular excretion) in the enterohepatic circulation, although the detailed molecular mechanism is unknown.

The current findings demonstrate that LD-fed *Elovl6*^−/−^*Ldlr*^−/−^ mice are more susceptible to gallstone formation as a result of an alteration in hepatic cholesterol metabolism that promotes hepatic free cholesterol secretion into bile, coupled with alterations in hepatic BA metabolism. The net result of these complex adaptations is a striking increase in the formation of cholesterol crystals. In the gallbladder bile, the solubility of cholesterol is maintained by the balance among cholesterol, bile salts and phospholipids^27^,^43^. The increased biliary concentration of cholesterol in *Elovl6*^−/−^*Ldlr*^−/−^ mice resulted in supersaturation, precipitation and crystallization of cholesterol. The FA compositional changes in the PL fraction might also affect the susceptibility of LD-fed *Elovl6*^−/−^*Ldlr*^−/−^ mice to gallstones. Several of these conclusions and their underlying mechanisms warrant further examination.

In conclusion, this study demonstrates that Elovl6 plays a crucial role in the development and progression of steatohepatitis through the regulation of FA, cholesterol and BA metabolism. These findings are an important step forward in understanding the pathophysiology of NASH and the contribution and interaction of Elovl6 in hepatic inflammation associated with hepatic lipid accumulation. Further studies are needed to determine the exact contribution of Elovl6 to the risk of developing NASH and may provide a basis for the development of alternative therapeutic strategies and markers for diagnostic tests.

## Methods

### Animals and diets

*Elovl6*^−/−^ mice (C57BL/6 background) were generated as described previously^16^. *Ldlr*^−/−^ mice (C57BL/6J background) were purchased from the Jackson Laboratory. *Elovl6*^−/−^ mice were crossed with *Ldlr*^−/−^ mice to produce *Elovl6*^−/−^*Ldlr*^−/−^ mice. The mice were housed in a pathogen-free barrier facility with a 12 h light/dark cycle and were given free access to food and water. Beginning at 11–16 weeks of age, mice were fed either a standard laboratory rodent chow diet (SD) (MF; Oriental Yeast, Tokyo, Japan) or an LD (16.5% fat, 1.25% cholesterol, 0.5% cholic acid (CA); F2HFD1; Oriental Yeast) for 4 weeks [n = 13 *Ldlr*^−/−^ mice fed SD (n = 5 of 11-week-old and n = 8 of 16-weeks-old), n = 8 *Elovl6*^−/−^*Ldlr*^−/−^ mice fed SD (n = 3 of 11-week-old and n = 5 of 16-week-old), n =12 *Ldlr*^−/−^ mice fed LD (n = 5 of 11-week-old and n = 7 of 16-weeks-old), and n = 13 *Elovl6*^−/−^*Ldlr*^−/−^ mice fed LD (n = 6 of 11-week-old and n = 7 of 16-week-old)]. Details of the LD are shown in Table S1 online. Age-matched male mice were used for all experiments. Mice were sacrificed during the light phase after food deprivation for 4 h. Plasma samples were collected from the post-orbital plexus. Tissues were isolated immediately, weighed and stored in liquid nitrogen. All animal husbandry and animal experiments complied with the guidelines of the University of Tsukuba’s Regulations of Animal Experiments and were approved by the Animal Experiment Committee of the University of Tsukuba.

### Plasma analysis

Plasma TC, TG, TBA and ALT were determined using commercially available assay kits (Wako Pure Chemicals, Tokyo, Japan). For the lipoprotein distribution analysis, plasma samples were analysed using an upgraded HPLC technique as previously described (Skylight Biotech Inc., Akita, Japan)^44^.

### Gallbladder bile and gallstone analysis

After 4 h fasting, animals were sacrificed and cholecystectomies were performed. The gallbladder was punched at the fundus to collect gallbladder bile. Bile samples were collected and immediately analysed by polarizing light microscopy for the presence of cholesterol monohydrate crystals.

### Hepatic lipid analysis

Total lipids were extracted from liver tissues using Folch solution (chloroform-methanol, 2:1 v/v)^45^, dried and dissolved in 2-propanol. Hepatic lipid extracts were assayed for TC and TG levels using commercial assay kits for blood cholesterol and TG determination (Wako Pure Chemicals).

### Hepatic total bile acid analysis

Hepatic TBAs were extracted from frozen tissue by homogenization in 75% ethanol and incubation at 50 °C for 2 h as previously described^46^. The extracted supernatants were assayed using a commercially available assay kit (Wako Pure Chemicals).

### Fatty acid composition of liver

Total lipids in liver were extracted using Bligh–Dyer’s procedure^47^, and TG and esterified cholesterol were separated on 500 mg silica columns (Supelclean PSA SPE Tube; Sigma-Aldrich Japan, Tokyo, Japan). The lipid fractions in each sample were methyl-esterified and the relative abundance of each FA was quantified by gas chromatography (SRL, Inc., Tokyo, Japan)^48^.

### Histology

Livers were removed, fixed in 10% neutral buffered formalin, embedded in paraffin and cut into 4-μm-thick sections for subsequent H&E staining.

### RNA extraction and quantitative real-time PCR

Total RNA was extracted from livers using Sepasol reagent (Nacalai Tesque, Kyoto, Japan) and was reverse-transcribed using the PrimeScript RT Master kit (Takara Bio Inc., Shiga, Japan) according to the manufacturer’s protocols. qPCR was performed using SYBR Premix Ex Taq (Takara Bio Inc.) and specific primer sets with the Thermal Cycler Dice Real Time System Single (Takara Bio Inc.). Primer sequences for *Acat2, Nr1h3, Ces3a, Ces3b, Nr1h4, Nr5a2, Nr0b2, Cyp7a1, Cyp8b1, Cyp27a1, Cyp7b1, Slc10a1, Slco1a1, Abcb11, Abcc2, Abcc1, Abcc3, Abcc4, Slc51b, Cd14 and Cybb* are summarized in Supplementary Table S2 online. Other qPCR primers used have been described previously^16^,^23^,^49^. The expression levels of mRNA were normalized to those of peptidylprolyl isomerase A (*Ppia*) mRNA.

### Immunoblotting

Immunoblotting was performed as described previously^16^. Protein isolated from whole livers was loaded onto 10% sodium dodecyl-sulphate polyacrylamide gel electrophoresis gels and transferred to polyvinylidene difluoride membranes (Millipore). The membranes were probed with anti-CYP7A1 (Santa Cruz Biotechnology, Dallas, TX), CYP27A1, SLC10A1, α-SMA (Abcam, Cambridge, UK) and phospho-JNK, JNK, glyceraldehyde 3-phosphate dehydrogenase (GAPDH) (Cell Signaling Technology, Denvers, MA), followed by horseradish peroxidase-conjugated anti-mouse or rabbit IgG (Cell Signaling Technology). Immune complexes were visualized using enhanced chemiluminescence (GE Healthcare).

### Statistical analysis

Results are expressed as mean ± SEM. Data between groups were analysed by Student’s *t*-test. Differences were considered significant at *P* < 0.05.

## Additional Information

**How to cite this article**: Kuba, M. *et al.* Absence of *Elovl6* attenuates steatohepatitis but promotes gallstone formation in a lithogenic diet-fed *Ldlr*^−/−^ mouse model. *Sci. Rep.* **5**, 17604; doi: 10.1038/srep17604 (2015).

## Supplementary Material

Supplementary Information
