# Supplementary material for: Absence of Elovl6 attenuates steatohepatitis but promotes gallstone formation in a lithogenic diet-fed Ldlr−/− mouse model
Source: Sci Rep. 2015 Dec 1;5:17604. doi: 10.1038/srep17604 (PMC4664962; doi:10.1038/srep17604)
Supplement: Supplementary Information [file srep17604-s1.pdf]

Supplementary Information

Absence of *Elovl6* attenuates steatohepatitis but promotes gallstone formation in a lithogenic diet-fed *Ldlr*$^{-/-}$ mouse model

Motoko Kuba, Takashi Matsuzaka, Rie Matsumori, Ryo Saito, Naoko Kaga, Hikari Taka, Kei Ikehata, Naduki Okada, Takuya Kikuchi, Hiroshi Ohno, Song-iee Han, Yoshinori Takeuchi, Kazuto Kobayashi, Hitoshi Iwasaki, Shigeru Yatoh, Hiroaki Suzuki, Hirohito Sone, Naoya Yahagi, Yoji Arakawa, Tsutomu Fujimura, Yoshimi Nakagawa, Nobuhiro Yamada, and Hitoshi Shimano

Supplementary Table S1

The Composition of the Lithogenic Diet (LD)

Composition	%
Cacao butter	7.500
Cholesterol	1.250
Cholate	0.500
Milk casein	7.500
Cellulose	1.250
Vitamin mix	1.000
Mineral mix	1.000
Sucrose	1.625
Glucose	1.625
Dextran	1.625
Choline chloride	0.125
Lard	3.000
CRF-1	72.000
Total	100.000

Supplementary Table S2

Sequence information for the quantitative Real-time PCR analysis

**Gene name**	**Forward primer (5' to 3')**	**Reverse primer (5' to 3')**
Acat2	CCCGTGGTCATCGTCTCAG	GGACAGGGCACCATTGAAGG
Nr1h3	AGCAACAGTGTAACAGGCGCT	ACGATGGCCAGCTCAGTAAAGT
Ces3a	ACTGGGACCTCTTCGGTTCT	GGTGAATCTGCTGTTGCTCA
Ces3b	CACAGACCGCATGGTAAATG	TTGATGCTGGCATCTCTCAC
Nr1h4	CTCTGCTCACAGCGATCGTC	CACCGCCTCTCTGTCCTTGA
Nr5a2	ATACAAACTCCCGCTGATCG	AAGTCGCGTTCAACAACCTC
Nr0b2	CGATCCTCTTCAACCCAGATG	AGGGCTCCAAGACTTCACACA
Cyp7a1	GCTGAGAGCTTGAAGCACAAGA	TTGAGATGCCCAGAGGATCAC
Cyp8b1	CTAGGGCCTAAAGGTTCGAGT	GTAGCCGAATAAGCTCAGGAAG
Cyp27a1	CCAGGCACAGGAGAGTACG	GGGCAAGTGCAGCACATAG
Cyp7b1	AGCCGATTATCAGCGAAAGCC	GCATCCAAAGGTTTGCCTTGT
Slc10a1	AGGGGGACATGAACCTCAG	TCCGTCGTAGATTCCTTTGC
Slco1a1	ACTCCCATAATGCCCTTGG	TAATCGGGCCAACAATCTTC
Abcb11	CAATGTTCAGTTCCTCCGTTCA	TCTCTTTGGTGTTGTCCCCATA
Abcc1	GATGGCTCCGATCCACTCT	AGGTAGAAACAAGGCACCCA
Abcc2	TCCAGGACCAAGAGTTTGC	TCTGTGAGTGCAAGAGACAGGT
Abcc3	TGAAGACTGCACCGTACTGAC	AGAAACCCTTGGAATGCATC
Abcc4	AGCTTCAACGGTACTGGGATA	TCGTCGGGGTCATACTTCTC
Slc51b	AGAGAAAGCTGCAGCCAATG	CCAGGACCAGGATGGAATAA
Cd14	GAAGCAGATCTGGGGCAGTT	CGCAGGGCTCCGAATAGAAT
Cybb	TTGGGTCAGCACTGGCTCTG	TGGCGGTGTGCAGTGCTATC

Supplementary Figure S1

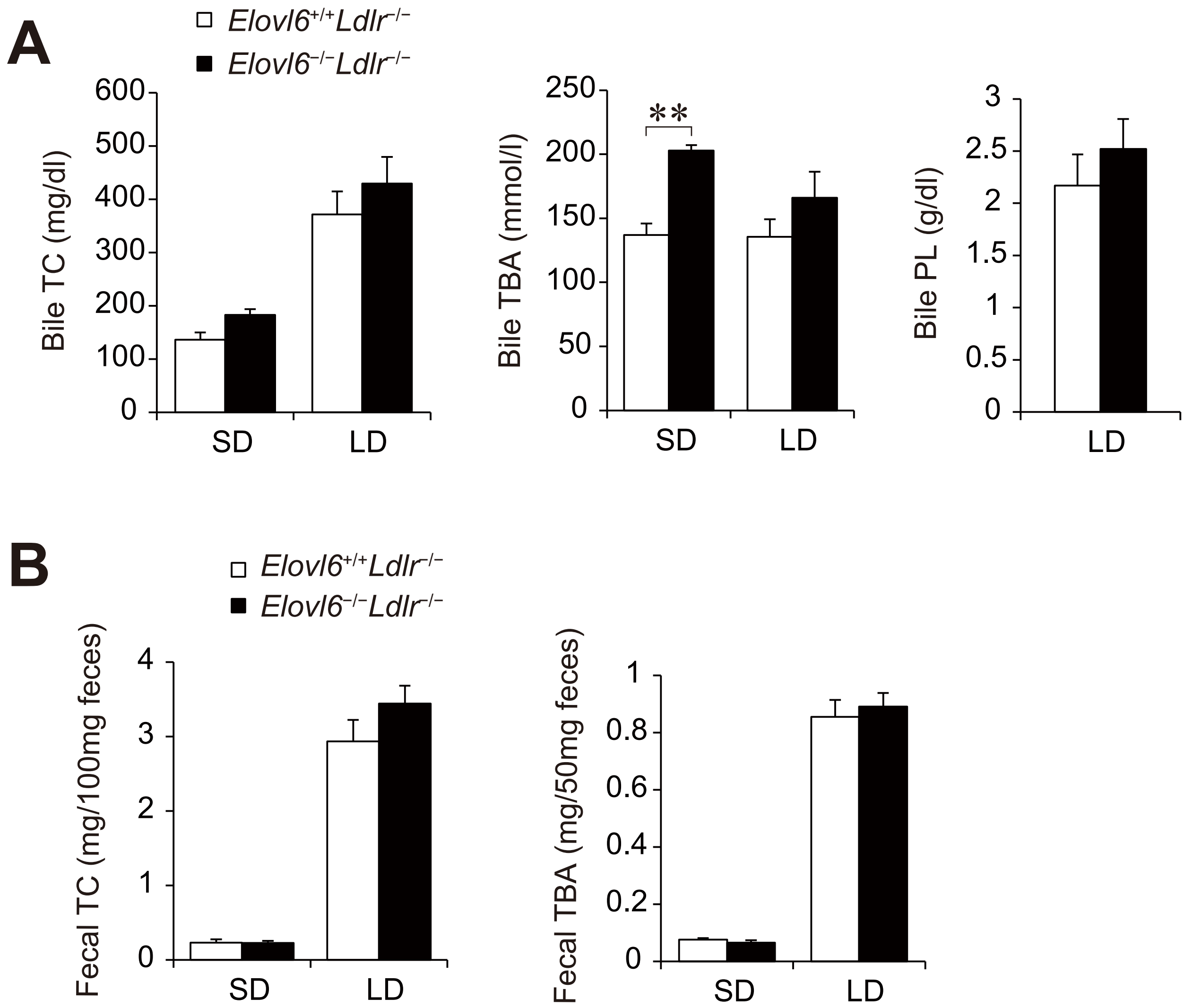

Supplementary Figure S2

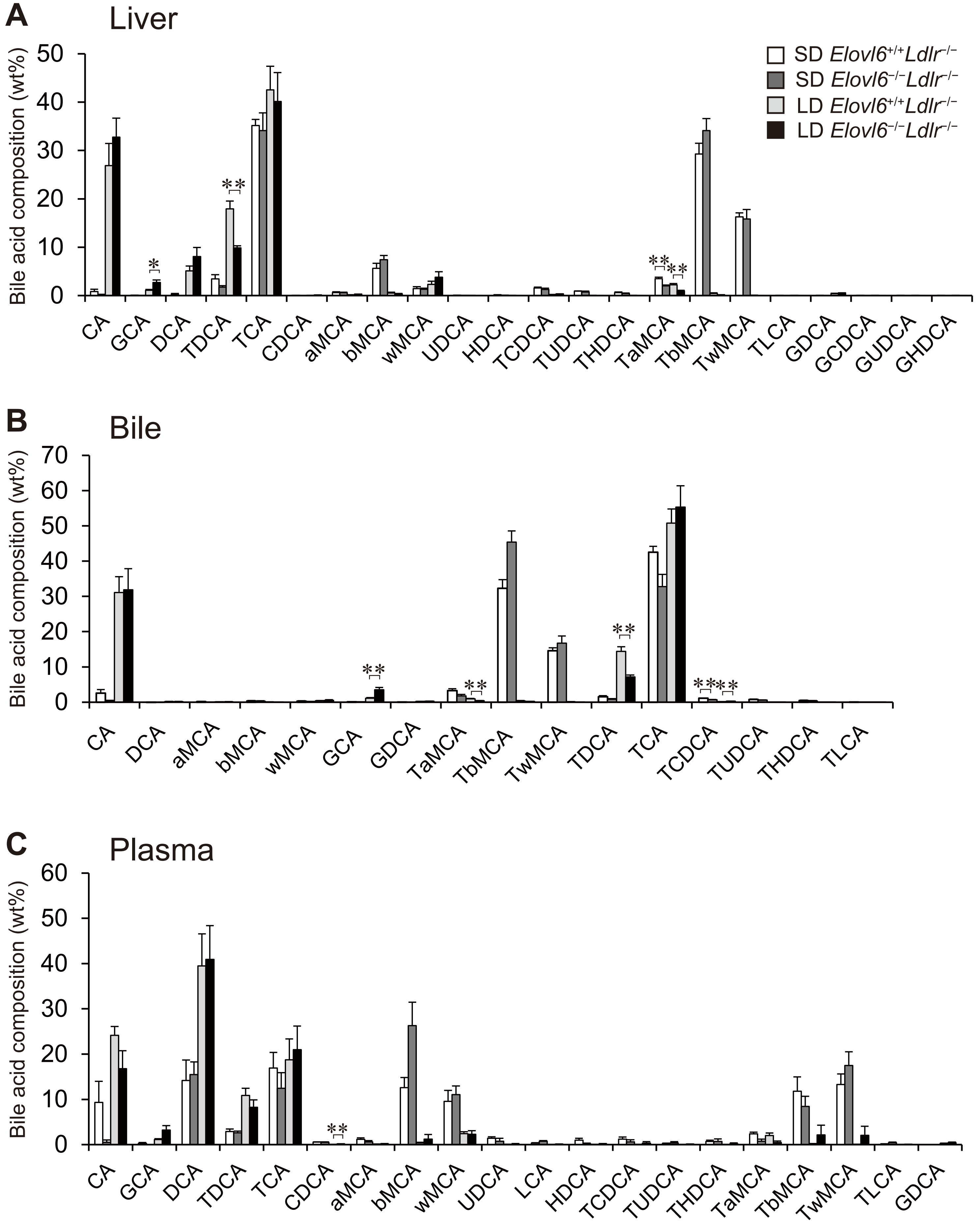

Supplementary Figure S3

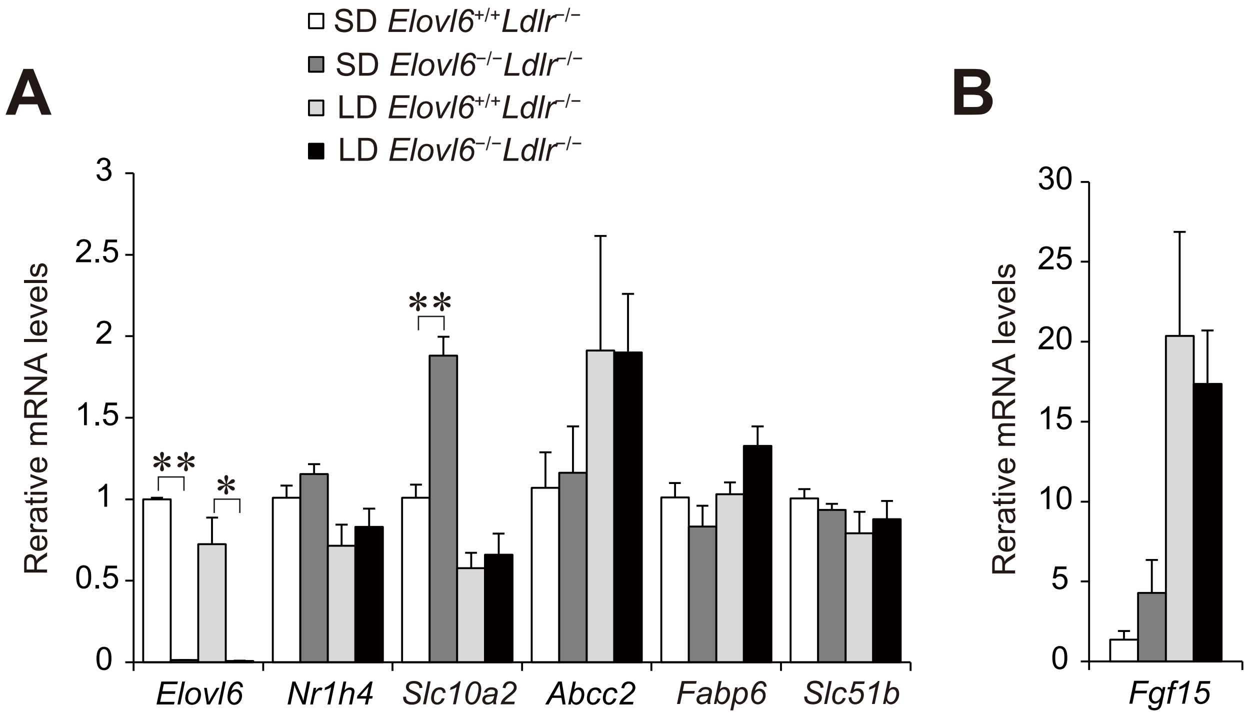

Supplementary Figure S4

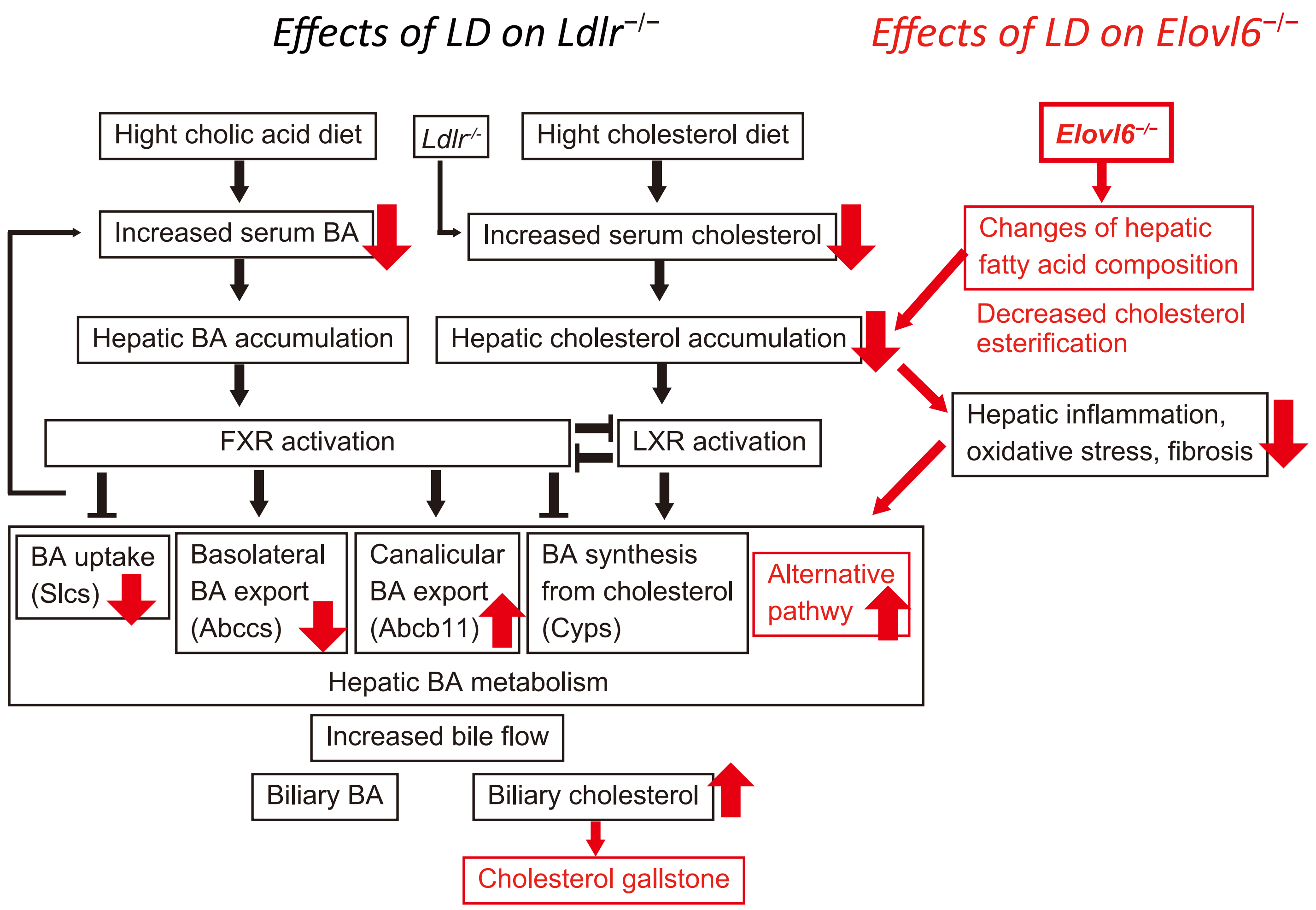

Supplementary Figure Legends

Supplementary Figure S1. Bile and fecal lipid profiles of LD-fed *Ldlr*$^{-/-}$ mice lacking Elovl6. (A) Bile total cholesterol (TC), total bile acid (TBA), and phospholipid (PL) levels (n = 3–8 per group) and (B) fecal TC and TBA levels (n = 7–10 per group) of *Elovl6*$^{+/+}$*Ldlr*$^{-/-}$ and *Elovl6*$^{-/-}$*Ldlr*$^{-/-}$ mice fed a SD or a LD for 4 weeks.

Supplementary Figure S2. Bile acid composition in (A) liver, (B) bile, and (C) plasma in *Elovl6*$^{+/+}$*Ldlr*$^{-/-}$ and *Elovl6*$^{-/-}$*Ldlr*$^{-/-}$ mice fed a SD or a LD for 4 weeks (n = 3–9 per group). individual BA species were examined by LC/MS. CA, cholic acid; GCA, glycocholic acid; DCA, deoxycholic acid; TDCA, taurodeoxycholic acid; TCA, taurocholic acid; CDCA, chenodeoxycholic acid; αMCA, alpha-muricholic acid; βMCA, beta-muricholic acid; ωMCA, omega-muricholic acid; UDCA, ursodeoxycholic acid; HDCA, hyodeoxycholic acid; TCDCA, taurochenodeoxycholic acid; TUDCA, tauroursodeoxycholic acid; THDCA, taurouhyodeoxycholic acid; TαMCA, tauro-α-muricolic acid; TβMCA, tauro-β-muricolic acid; TωMCA, tauro-ω-muricolic acid; LCA, lithocholic acid; TLCA, taurolithocholic acid; GDCA, glycodeoxycholic acid; GCDCA, glycochenodeoxycholic acid; GUDCA, glycoursodeoxycholic acid; GHDCA, glycouhyodeoxycholic acid. $* P < 0.05$, $** P < 0.01$.

Supplementary Figure S3. Quantitative real-time PCR (qPCR) analysis of genes involved in bile acid homeostasis in the ileum. *Elovl6*$^{+/+}$*Ldlr*$^{-/-}$ and *Elovl6*$^{-/-}$*Ldlr*$^{-/-}$ mice were fed a SD or a LD for 4 weeks and sacrificed following 4 h of food deprivation (n = 4–7 per group). qPCR analysis of genes for (A) *Elovl6*, *Nr1h4*, *Slc10a2*, *Abcc2*, *Fabp6*, *Slc51b* and (B) *Fgf15*. ∗ $P < 0.05$, ∗∗ $P < 0.01$.

Supplementary Figure S4. Proposed mechanism of Elovl6-mediated metabolic alteration in LD-induced liver injury and gallstone formation.

Supplementary Methods

Individual bile acid analysis by Liquid Chromatography/Mass Spectrometry

We determined individual bile salt compositions by liquid chromatography/mass spectrometry. BA standards were purchased from Sigma. For liver samples, approximately 100 mg of liver was homogenized in 2.5 volumes of H_2O. d4-CA (Internal standard: IS) and 400 µl of ice-cold ethanol were added to 200 µl liver homogenate. Samples were vortexed, centrifuged, and the supernatants were evaporated before being reconstituted in a 200 µl of 50:50 solution of ethanol and water. For plasma samples, 450 µl of ice-cold EtOH was added to 50 µl plasma-spiked with IS, vortexed, and centrifuged. The supernatants were evaporated and reconstituted in a 100 µl of 50:50 solution of ethanol and water. Bile samples were diluted 100- and 2000-fold with a 50:50 solution of ethanol and water, and IS was added. The LC system used was a HPLC (Gilson) equipped with an autoinjector. The HPLC was connected to a TSQ Quantum Ultra (Thermo Scientific). Individual BAs were eluted with gradient at a 0.2 ml/min flow rate for a mobile phase A (10 mM $AcONH_4$ in H_2O) and mobile phase B (10 mM $AcONH_4$ in a 23:17 solution of CH_3CN and ethanol). The samples were eluted with 80% mobile phase A and 20% mobile phase B for an initial 5 min after injection, and then with a linear gradient of mobile phase B of 20% to 75% over 55 min, which was held for 5 min. The injection volume of all samples was 5 µl. Quantitative analysis was performed in negative ionization mode using the selected reaction

monitoring transitions specific for each BA.
